# Influence of Student Selected Components on Medical Career Choice

**DOI:** 10.15694/mep.2017.000191

**Published:** 2017-10-26

**Authors:** Ana-Catarina Pinho-Gomes, David Taylor, Simon Riley

**Affiliations:** 1 University of Liverpool; 2 University of Edinburgh

**Keywords:** Student selected components, Career choice, Special study modules, Medical careers

## Abstract

This article was migrated. The article was marked as recommended.

***Background and aims**:* Student selected components (SSC) offer a privileged opportunity for students to gain a deeper insight into areas of their personal interest, including potential careers. How different SSC programmes influence future career choice remains uncertain. The aims of this study were to investigate (1) how SSC programmes in British medical schools offer career exploration and (2) whether the experience of SSCs during medical school influences medical career choice.

***Methods**:* Two online surveys were sent to SSC leads in medical schools and to Foundation Year 2 doctors in the UK. This information was complemented with data available on medical school websites.

***Results and conclusions**:* A wide diversity of SSCs programmes is provided by medical schools across the UK, with variable compliance with GMC recommendations regarding career exploration. SSCs seem to play a paramount role in shaping career preferences during medical school and to exert a powerful influence on future career decisions. Therefore, it is imperative to design SSC programmes that allow students to explore several career pathways, including both medical and alternative careers, so that they can make informed decisions and hence avoid the detrimental consequences of inadequate career choices.

## Introduction

Workforce planning is a central issue for service provision and has consequences for medical education. Health services need a supply of medical graduates willing to train in all specialties, in the right proportions and, crucially, in the right places, to meet healthcare needs. The role of medical schools is to form a pool of graduates who are not only competent and professional doctors but also match the contemporary needs of the healthcare service. Therefore, informing career choice and helping with career decision-making are increasingly important roles that medical schools are expected to fulfil. Students with inappropriate career aspirations, often based on inaccurate or incomplete understanding of each medical specialty, can have a detrimental effect during subsequent medical training because career decisions are made early and difficult to reverse ( Cleland J et al. 2016; Heikkil 2016; van Wulfften Palthe et al. 2016). These inadequate career choices can lead to distress, possible reallocation, or drop-out ( Dodson and Webb 2005).

Medical career choice results from a rational, albeit subjective, match of perceptions of specialty characteristics against a rank of individual needs ( Querido et al. 2016). Specialty preferences are often present upon entry to medical school ( Cleland J et al. 2012; Ibrahim et al. 2014). However, they usually change as a student progresses towards graduation, and the majority eventually specialise in an area different from that early preference ( Kaur et al. 2014). The impact of medical schools on career preferences results from a constellation of factors, such as institutional culture, faculty values, curriculum content and format, learning activities and opportunities to explore alternative career pathways ( Coffeng and Visscher 2009; Kuhnigk et al. 2009; Cleland JA et al. 2014). Therefore, medical schools have a duty to provide opportunities for career exploration ( Querido et al. 2016), which aim to encourage students to gain appropriate clinical experiences in different specialties, to discover and establish their personal career needs, and the matching of career needs to specialty perceptions.

The SSCs, which are an intrinsic curricular component in all medical schools across the UK ( Riley 2009), are excellent opportunities for in-depth learning and exploring specialties that are either covered superficially or completely overlooked by the core curriculum ( Cave et al. 2007). This role of SSCs in providing career exploration was recognised in the second edition of Tomorrow’s Doctors, which established that SSCs ‘must allow students to consider career paths’ ( GMC 2003), and gaining a deeper insight into some medical specialties can be the chief motivation underlying student’s choice of SSCs ( O’Tuathaigh et al. 2012). However, it remains uncertain whether SSCs encourages students to become generalists or specialists, affects their future career aspirations, or helps make the complex decision on which medical career to commit themselves to. Therefore, this study aimed to characterise the different SSC programmes available across the UK regarding career exploration and assess whether students’ experience of SSCs influences medical career choice.

## Methods

### Study design

This cross-sectional study used two electronic questionnaires sent to foundation year 2 (FY2) doctors and SSC leads in British medical schools to analyse the impact of SSCs on medical career choice.

### Setting, population and instrument

Post-graduate training in the UK starts with a 2-year foundation programme, which is followed by specialty training (either run-through or via a 2-stage application programme). Applications happen in the first months of the second foundation year and thus career decision making is mainly based on medical school and early post-graduate training experiences, which depending on the programme may or may not be in their specialty of interest.

This study invited Foundation Schools across the UK to send an electronic survey to their FY2 doctors about the influence of SSCs on career choice. All SSC leads in medical schools across the UK were also invited to answer an electronic questionnaire about the characteristics of the programme they coordinate and how they perceive it influences medical career choice. Both the questionnaires were developed using the software available online on Google Forms (Supplemental data). There was a mix of multiple-choice and open-questions to justify the answers and allow participants to freely convey their opinion. No demographic or personal identifiable information were included.

The information provided by SSC leads was complemented with the data available on medical school websites, particularly when SSC leads refused to participate in the study.

All participants were sent an information leaflet with a detailed description of the study.

### Data analysis

Qualitative data was analysed using thematic analysis ( Braun and Clarke 2006) to identify patterns and common themes in the answers to open questions. Emerging themes were related to quantitative data. The most relevant themes were selected based on frequency and emphasis.

Quantitative data was statistically analysed. Categorical variables were presented as counts and percentages.

### Ethical considerations

This project conforms to the Declaration of Helsinki and was approved by the Ethics Committee of the University of Liverpool. The permission was accepted as proof of review by the medical and foundation schools.

## Results

### The students’ perspective: FY2 doctor’s questionnaire

A total of 103 FY2 trainees answered the questionnaire (amongst around 7800 FY2 doctors in the UK). This low response rate (1.4%) was due to the fact that many foundation schools refused to send the link to the questionnaire to their trainees to avoid ‘survey fatigue’. As most schools did not reply accepting or declining to participate in this study, it was impossible to know whether schools sent the survey to their trainees.

The participants came from 24 out of 33 medical schools. When enquired about their future career plans, most of them were planning to either continue medical training (62 out of 103) or take a year out (35 out of 103), with a minority wanting to pursue a career in military medicine (2 out of 103) or to leave medicine and find an alternative career (4 out of 103). Of those interested in continuing medical training, the most common pathways were core medical or surgical training and acute care common trunk (16, 15 and 19 out of 103, respectively). However, 17 out of 103 were unsure about their future specialty.

**Figure 1.**
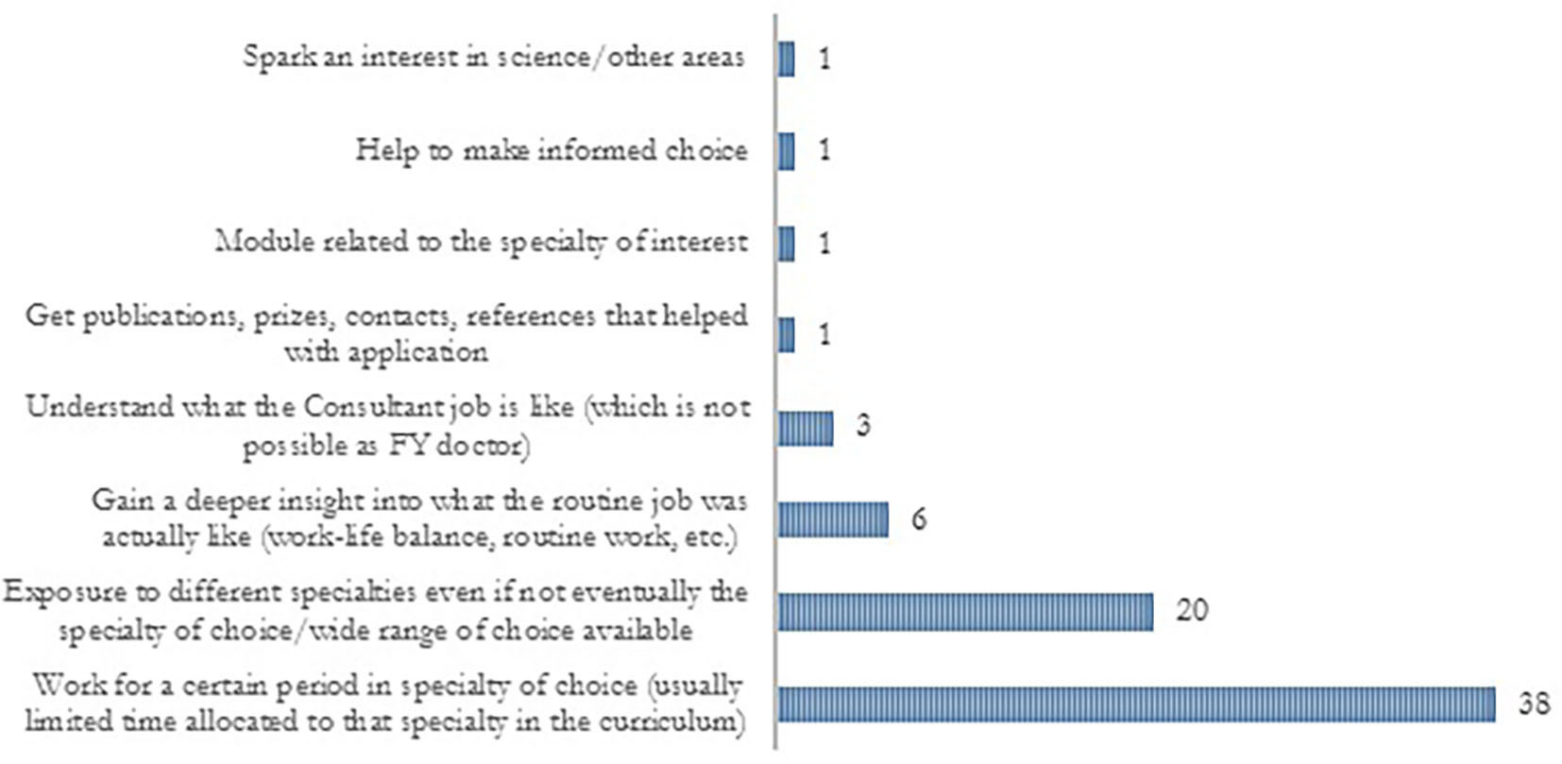
Different ways in which SSCs provided career exploration according to FY2 doctors

**Figure 2.**
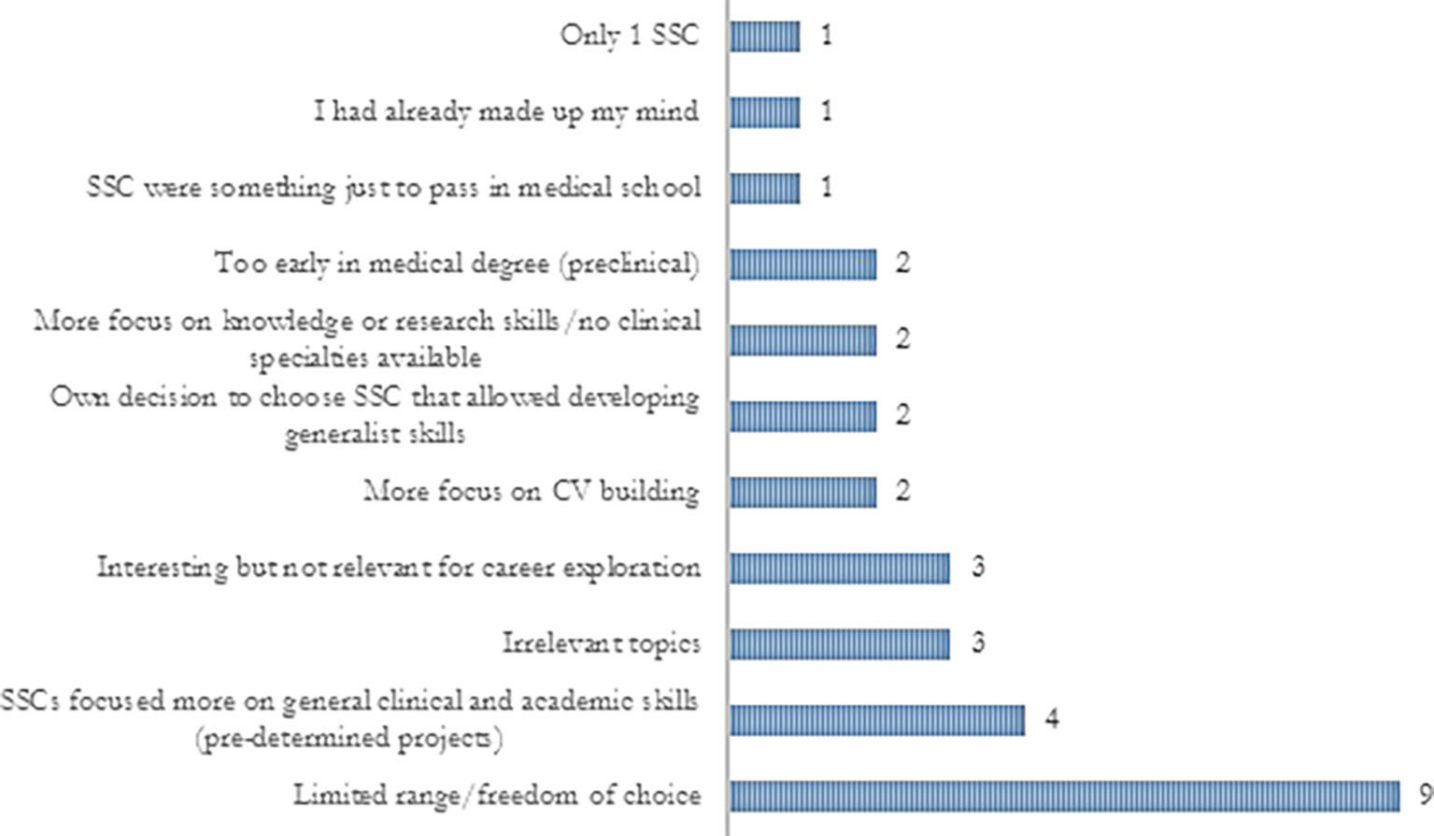
FY2 doctors’ explanations on how SSCs did not provide career exploration

**Figure 3.**
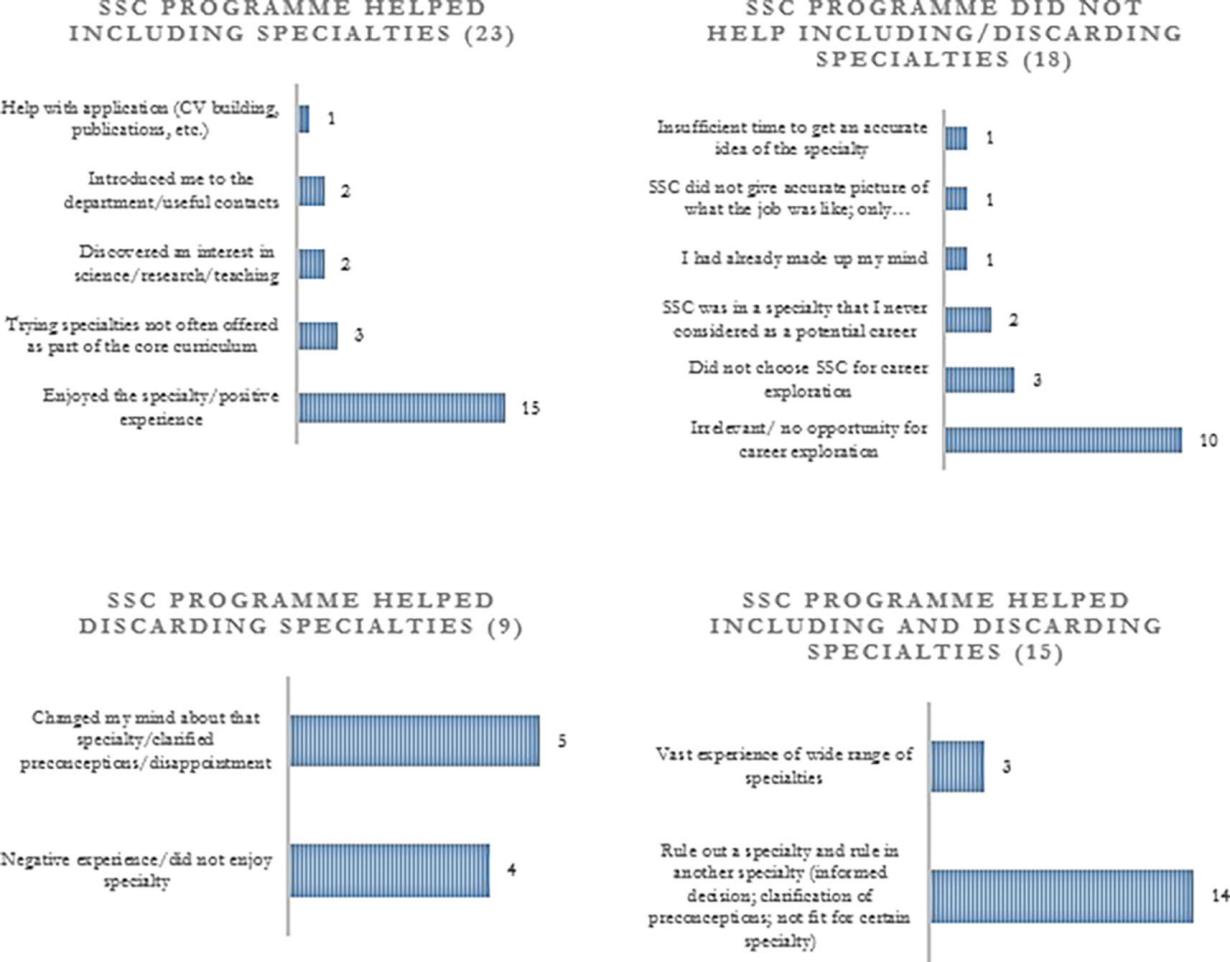
Different ways in which SSC programmes allowed students to explore career options; numbers in brackets represent the total number of students in each category

**Figure 4.**
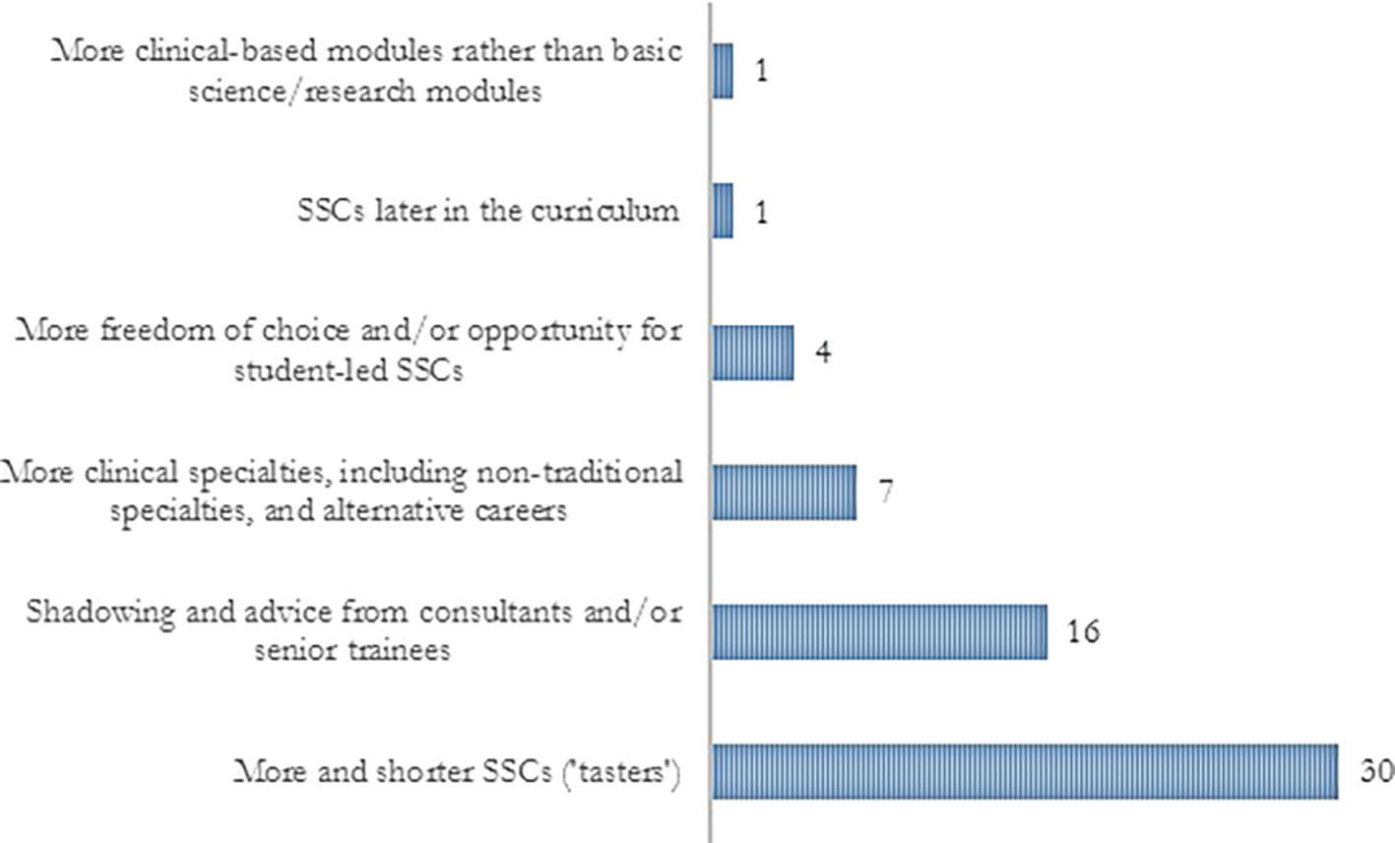
FY2 trainees’ recommendations on how SSCs could provide better opportunities for career exploration

About 75% of the trainees agreed that SSCs allowed them to explore career options and they identified several ways in which career exploration was provided ( Figure 1 and 2). SSC programmes were useful for both including and discarding specialties and the underlying reasons are outlined in Figure 3. Although most trainees agreed that SSCs provided some degree of career exploration, 75% mentioned that they would have liked to have further opportunities. Their suggestions are summarised in Figure 4.

### The medical school and tutors’ perspective: SSC leads questionnaire and online search

The SSC programmes available in British medical schools vary with regards to time commitment, distribution throughout the curriculum, degree of choice, as detailed in Supplemental Table 1. Career exploration was considered an intended learning outcome of the SSC programme in 11 out of 33 schools.

Ten out of the 14 SSC leads who replied to the online survey agreed that exploring career options was one of the objectives of their SSC programme. Furthermore, seven out of those 10 SSC leads considered that the programme was effectively providing opportunities for career exploration because students could experiment different clinical specialties and gain a deeper insight into potential careers, sometimes even by designing their own SSCs. Nevertheless, only five out of 14 SSC leads considered that the SSC programme exerted a significant influence on future career choice, with the remaining showing uncertainty as the extent of that influence would vary depending on students’ choice and commitment. Most SSC leads indicated that their SSC programme would be unable to provide more opportunities for career exploration due to time constraints, lack of resources and competing interest, for instance with core curriculum content.

## Discussion

This cross-sectional study revealed that most FY2 trainees were provided with a degree of career exploration by SSC programmes but most considered opportunities were insufficient. In keeping with this, the current scenario of SCC programmes offered by medical schools across the UK is very heterogeneous and compliance with GMC recommendations regarding career exploration is variable. Therefore, FY2 doctors recommended that SSC programmes should include shorter and more varied placements, with more freedom of choice to allow students to experiment a vast range of potential specialties, including also alternative careers.

### FY2 doctors’ view

Exposure to a certain specialty and/or locality seems to exert a paramount influence on students’ career choice ( Nichols et al. 2004). Students’ perceptions evolve throughout medical school as they experience a broader range of specialties, particularly those that are associated with less public exposure and/or contact (for example, anaesthetics). This reinforces the importance of high quality SSC programmes, which provide students with a wide breath of medical specialties, particularly those commonly left aside by the core curriculum.

Most trainees were interested in pursuing ‘generalist’ pathways, like core surgical and core medical training and acute care common stem, which might reflect their uncertainty about which specialty they want to pursue, their inability to commit to a lifelong career at such an early stage, or that they wished to keep their options open, and gain more insight as their training continues to progress. Furthermore, a considerable proportion of them was unsure about their preferred career. Taken together, those findings reinforce the importance of career exploration in medical school to allow foundation doctors to make informed decisions about their future.

Although most FY2 trainees agreed that SSCs did provide opportunities for career exploration, some were adamant that SSCs were useless in this regard. This appeared to be associated with insufficient choice and research-related SSCs. Tutors and students’ perceptions on learning outcomes in SSC programmes often do not overlap ( Murphy et al. 2008), particularly when student-led SSCs are not available ( Murphy et al. 2009). Therefore, better clarity in the definition and communication of expected learning outcomes, including career exploration, may be warranted to ensure better alignment of students and tutors’ intensions in SSCs.

It was clear that SSCs were useful both to include and rule out career choice of specialties, depending on the quality of students’ experiences. Indeed, positive experiences helped confirming students’ preference for a certain specialty or to discover new interests or specialties that were outside the core curriculum. Students excluded specialties because they had a negative experience, they realised that they were not suited for that type of work or professional lifestyle. By immersing students in the clinical environment, SSCs may have provided a more realistic perception that may be counter to a previously more positive experience during a core rotation. Although career exploration is not necessarily confined to SSC programmes, core placements often fail to provide adequate exposure, which may result in skewed perspective on the routine work in a certain specialty. The less formal design of SSCs and the more exploratory view expressed in the learning outcomes, experience undertaken, and the assessment, may foster closer relationships between students and clinical staff, who can offer valuable guidance and advice that would be difficult to gather from other sources. Furthermore, the high number of students in core rotations commonly restricts access to outpatient clinics, multidisciplinary meetings, theatre, endoscopy, which make the bulk of a Consultant job ( Alyusuf 2012). Therefore, core rotations, albeit essential to gain generic skills and knowledge that are central to the formation of any doctor, rarely provide a true perspective of the routine work in a certain medical specialty ( Newbegin et al. 2007). As SSCs are typically more flexible, students may have a different mind-set that promotes reflection and self-awareness, both of which are crucial for career decision making ( Stark et al. 2005).

A consistent recommendation by FY2 trainees was to increase the diversity and decrease the duration (one to two weeks) of placements available in SSC programmes, to allow students to ‘taste’ different specialties and/or alternative careers. Although these ‘taster weeks’ would be beneficial for exposing students to a wide diversity of specialties and environments, it is arguable that such a limited and brief experience would be meaningless. A short glimpse of something as complex as a medical specialty would more likely generate biased views than a true understanding of the work and work-life balance, which are often the main focus of students’ attention. The opportunities for career exploration after medical school are shrinking with doctors having to apply for subsequent training at an increasingly earlier stage ( GMC 2011) and this may explain why trainees would have liked more opportunities for trying different careers during undergraduate training. However, reconciling career exploration with other competing pressures in an already overloaded curriculum is a challenge for most medical schools. The recognition of this issue by the GMC lead to the recent release of new standards and guidance for postgraduate curricula and assessment. The new standards move towards a high-level outcomes approach to learning, giving doctors more freedom and choice to change specialties as their interests in medicine develop ( GMC 2017).

### Medical school and SSC leads’ view

Although most SSCs agreed that career exploration was a learning outcome of their SSC programme and it was effectively delivered, the results did not match with the answers of the FY2 trainees. Indeed, some of the FY2 trainees who had graduated in the medical schools whose SSC leads stated that SSCs provided career exploration, denied to have had opportunities for that. A potential explanation is the lack of alignment of learning outcomes perceived by tutors and students ( Murphy et al. 2008), which may require not only better communication but also offering students the possibility of designing and organising their own SSCs.

The reluctance to consider career exploration as an outcome of SSC programmes may be at least partially underpinned by the scepticism demonstrated by SSC leads about their impact on future career choices. Only 11 out of the 33 medical schools clearly mentioned that career exploration was an intended learning outcome of at least one of the SSCs, with a further 3 stating that it would depend on the specific SSC chosen by the student. The fact that some medical schools did not mention SSCs (or equivalent concept) in the curriculum and/or any other course information available online clearly demonstrated the lack of investment and commitment to those curricular elements. Although SSCs were introduced as a novel educational entity over 20 years ago, this study confirms the variable implementation that has been previously described ( Murdoch-Eaton et al. 2004). The underlying reasons are perhaps not completely understood, but lack of support and prescriptive guidance from the GMC are potential causes. However, Tomorrow’s Doctors ( GMC 2009) indicates that “SSCs support the core curriculum and must allow students to (..) consider potential career paths”. Therefore, career exploration cannot be overlooked when designing SSC programmes, particularly because the very nature of SSCs puts them in a privileged position to explore potential careers.

### Limitations

Although the survey was emailed to all Foundation Schools across the UK, some refused to participate in this study to prevent ‘survey fatigue’. This together with the small sample size due to the poor response rate precludes drawing definite conclusions about the true impact of SSC programmes on career choice. The possibility of unintended selection bias cannot be excluded. Therefore, the extent to which the findings of this study accurately represent the overall view of the entire population of FY2 trainees across the UK is uncertain. Nevertheless, consensual opinions, like the need for more diversity and freedom of choice, are probably worth considering in curriculum design. Furthermore, this cross-sectional study did not take into account that the opinion of FY2 doctors about career choice may have changed over time, retrospective assessment and recall bias, all of which can influence perceptions of the impact of previous experiences, including SSCs, on career decisions.

A strength of this study is that it included FY2 doctors who had attended 25 different medical schools. However, the small sample size of FY2 doctors meant that any subgroup analysis with their own medical school would not be appropriate. Therefore, although the total number of participants is small, the fact that a broad range of SSC programmes and medical schools are reflected in the study contributes to its generalisability. Finally, a detailed description of the different SSC programmes offered across the UK remains incomplete due to the limited amount of information available online for those schools whose SSC leads who did not respond to the questionnaire.

## Conclusion

There is a wide range in the learning outcomes, dedicated time, and organisation of SSC programmes available across the UK, with variable compliance with GMC recommendations regarding career exploration. Furthermore, SSCs are ideal for students to develop autonomy and self-awareness, which are essential to make wise decisions on future careers. The powerful influence of SSCs on students’ decisions and future career means that they should not be considered as a minor or supplementary part of the curriculum, and emphasises how carefully they should be designed and implemented. Only by providing adequate opportunities for in-depth career exploration it will be possible to avoid inappropriate career choices and their deleterious consequences for the individual and the healthcare system overall. The implementation of a successful SSC programme that includes career exploration as a main learning outcome presents its own challenges, with the ongoing issue of curriculum overload exacerbated by the rapid expansion of medical knowledge and technology.

## Take Home Messages

• There is a wide variety of SSC programmes across the UK, but career exploration features as a main learning outcome in a minority of them.
• Greater compliance with GMC guidelines regarding career exploration is not only desirable but also necessary in the current setting of British medical postgraduate training.
• SSC programmes are the ideal curricular component to provide career exploration, which include medical, and with flexibility non-medical or non-traditional careers.
• Students and FY2 doctors favour widening the breath of choice, even at the expense of shortening the duration of individual modules, particularly if self-designed SSCs are allowed.

## Notes On Contributors

Dr Ana-Catarina Pinho-Gomes is a specialist trainee in Cardiothoracic Surgery in the UK and has recently completed the MSc in Medical Education in the University of Liverpool.

Rev David CM Taylor is Director of SSCs and Director of the MSc in Medical Education at the University of Liverpool.

Dr Simon C Riley is Director of SSCs at the Edinburgh Medical School.

## Appendices

### Supplemental Table

**Table 1.** Summary of SSC programmes available in medical schools across the UK (data obtained from online questionnaire to SSC leads and medical school websites)

Medical Schools	N	Type	Duration and % timetable	Position	Choice ^j^	Learning Outcome ^g^
**Brighton and Sussex Medical School**	5+	Y 1-2: undertake individual studies and explore selected topics in depth, informed by the latest research Y3: clinical specialties or non-medical areas Y4: longitudinal research project Y5: student organised clinical experience	Not specified	Y1-5	Choice from list and self-proposed	Yes (Y5)
**Cardiff University**	4	Y2-3: Research Y4: Journal article Y5: Conference organisation; Elective	7-9w	Y2-5	Choice from list and self-proposed	Yes
**Hull and York Medical School**	5	Y1-2: Research-based (laboratory) Y3: Clinical-based including audit Y4: Longitudinal project Y5: Elective	Not specified	Y1-5	Choice from list and self-proposed	No
**Imperial College London**	2	Y6: Specialty choice module; Elective	3 and 8w	Y6	Choice from list and self-proposed	No
**Keele University**	6	Y1: Literature review Y2: Working in voluntary or statutory organisations Y3: Research, clinical or humanities based module Y4: Career exploration/shadowing Y5: Elective	Y1: 3w Y2: 8d Y3: 2x4w Y4: 4w Y5: 8w	Y1-5	Choice from list or self-proposed	Yes (Y3-4)
**King’s College London**	4	SSC in medical, scientific and non-medical subjects; scholarly project; or taster		Y2-3		Yes
**Newcastle University**	3	Medical and non-medical subjects	SSC: 6w Elective: 8w	Y3-4		
**Norwich Medical School**	2	SSC are part of the curriculum but no information available other than elective	SSCs: 4w Elective: 6w	Y4-5		
**Peninsula College of Medicine and Dentistry**	5	1 module per year split into themes, which depend on yearY5: Elective	Fixed time frame or longitudinal 2w to 1yElective: 8w(20% of timetable)	Y1-5	Choice from list or self-proposed	No
**Queen Mary University of London**	11	SSCs in any medically related field - clinical or nonclinical.Y5: Elective	2-5w modules and longitudinal SSCs Y2-5(16-17% of timetable)	Y1-5	Choice from list or self-proposed	Yes
**Queen’s University Belfast**	SSCs based in the community, clinical and research-based but no detailed information available					
**St Andrews Medical School ^**^**	1	Dissertation	10w	Y2	Choice from list	No
**St George’s University of London**	5	No information available other than Elective	6w	Y2-3; Y5		Yes
**The University of Edinburgh**	8	Y1: SSC1 patient safety group project Y2: (a) group project literature review and (b) group project on any topic Y3: SSC3 teamworking/solo Y4: (a) solo substantial research project (most frequently clinical audit) and (b) peer teaching Y5: (a) elective and (b) linkage module to FY1 job	1 year spread - all elements are longitudinal, not a pure block, embedded with teaching and /or clinical elements(20% of timetable)	Y1-5	Choice from list or self-proposed	Yes
**The University of Warwick**	3	Y1: SSC 1: taught module Y3: SSC 2: research project Y4: SSC3: Elective	SSC 1: 3h session over 10wSSC 2: 8 wElective: 6w(10% of timetable)	Y1, 3 and 4	Choice from list or self-proposed	No
**University College London**	6	Different types available. The final SSC must be form the preparation for practice category as it is post finals Y6: Elective	Y1-2: 4x 8 half days Y6: 2x4wElective: 8w	Y1-2 and 6	Mainly choice from list: self-proposal very limited	SSC-dependent
**University of Aberdeen**	1	Y1: Evidence-based medicine (literature review) Y5: Elective	Y1: 2w Y5: 8 w	Y1 and Y5	Choice from list	No
**University of Birmingham**	?	Non-modular required components/zero-credit (unclear) Y4: Elective	Elective: 8w	Year 1-5	Choice from list or self-proposed	Not specified
**University of Bristol**	5	Y1: Library project Y2: Group projects Y3-4: Individual placements Y5: Elective	SSCs: 4wElective: 8w(10-15% of timetable)	Y1-5	Choice from list or self-proposed	Yes (Y5)
**University of Cambridge***	1	Research-based (unclear)	6w	Y4		
**University of Dundee**	6+	Y1: longitudinal exercise Y2-3: modules Y4: longitudinal project Y5: elective and clinical SSC	2-4w modules or over a yearElective: 8w(25% of timetable)		Choice from list or self-proposed	Not specified
**University of Exeter**	12	Wide variety (about 300 different topics offered);short and intensive or longitudinal; academic and clinical Y5: Elective	2-3w or over a year Elective: 8w(20-25% of timetable)	Y1-4	Choice from list	SSC-dependent
**University of Glasgow**	5	Projects cover topics from the core curriculum as well as topics outside medicine including humanities and languages. Y3-4: Electives (x2)	SSCs: 5w Electives: 4w	Year 2-4	Choice from list or self-proposed	Not specified
**University of Lancaster**	3	SSM (3): evidence-based medicine (literature review)SAMP (2): different clinical specialties Y4: Elective	SSM: 4w SAMP: 7w Elective: 5w	Year 1-5	Choice from list	Yes
**University of Leeds**	No mention to SSC					
**University of Leicester**	5	Y5: Elective	Y3: 2x4w Y5: 2x8w Elective: 6w	Y3 and 5	Not specified	Yes
**University of Liverpool**	4	Y1: structured review Y2-3: laboratorial, audit and clinical projects Y5: elective	4w in total programmeElective: 5w(2% of timetable)	Y1-3 and 5	Choice from list	No
**University of Manchester**	8	Vary from academic outcome focused to career focused Y4: Elective	3-11 w(20% of timetable)	Y1-5	Choice from list or self-proposed	SSC-dependent
**University of Nottingham**	4	Initially clinical or research-based and later clinical-based Y5: Elective	SSM: variableElective: 6w	Y2, Y4-5	Choice from list	No
**University of Oxford**	4	Research/clinical/medicine& society Y6: Elective	17w in totalElective: 10w(8% of timetable)	Y3, 4 and 6	Choice from list or self-proposed	Yes
**University of Sheffield**	6+	Y1: SSC in medical ethics and law Y2: SSC in social accountability: voluntary work with patient or community groups Y3-4: Community-based and clinical audit Y4: Elective		Year 1-5	No or minimal choice	No
**University of Southampton**	5	Y1: 2 blocks of 2 SSCs Y3: clinical and non-clinical areas Y5: Elective and clinical SSC	Not specified	Year 1, 3 and 5	Choice from list but Y1 restricted to medical humanities and health improvement	Yes
**University of Swansea ^*^**		No mention to SSC Y4: Elective	Elective: 6w			Not specified

^*^ Graduate only

^**^ Undergraduate only

^j^ Extent to which students have freedom of choice in the programme

^g^ Career exploration is a learning outcome of the SSC programme as a whole or at least of part of it

### Questionnaire for Foundation Year 2 doctors

This survey aims to assess the impact of student-selected components in medical school on future career choices. The results will be freely disseminated, including through publication and presentation in scientific meetings. Completion indicates your consent for this analysis, distribution and publication of anonymised, grouped results. Individual responses remain anonymous. You will never be asked to provide any personally identifiable information. Your contribution is very appreciated.

Which medical school did you attend?

What was your type of entry?

• Undergraduate
• Graduate

Did you do an intercalated Hons / MSc?

Which foundation school are you working for?

What are you thinking of doing next year?

• Leaving medicine for an alternative career
  • Which alternative career are you thinking of and why?
• Taking a year out (locum work, travelling, voluntary work, etc.)
  • Please specify what your plans are:
• Continuing medical training
  • Which specialty are you thinking of?
    • Core medical training
    • Core surgical training
    • General Practice
    • Acute Care Common Stem (Anaesthetics, Emergency Medicine and Acute Medicine)
    • Run-through surgical specialty
    • Obstetrics and Gynaecology
    • Paediatrics
    • Academic Clinical Fellowship
    • Public Health
    • Psychiatry
    • Other. Which?

Did your SSCs provide opportunities to explore future career options?

• Yes
• No

Please justify your answer

Did your SSC help you to:

• Include specialties as potential career
• Discard specialties as potential career
• Both of the above
• None of the above

Please justify your answer

Would you have liked to have more opportunities to explore career options in medical school?

• Yes
• No

Please explain what these career opportunities may look like.

Thank you very much!

### Questionnaire for SSC leads

This survey aims to assess the implementation of student-selected components in medical schools across the UK and their impact on future career choice. The results will be freely disseminated, including through publication and presentation in scientiCc meetings. Completion indicates your consent for this analysis, distribution and publication of anonymised, grouped results. Individual responses remain anonymous. Your contribution is very appreciated.

In which medical school do you teach?

What is your professional background?

### SSC programme

Please describe the SSC programme in your school with regards to the following topics

Number

Short-answer text

Type

Short-answer text

Duration

Short-answer text

Percentage of the timetable that is dedicated to SSC

Percentage of the timetable that is dedicated to SSC

Position(s) in the overall programme

Choice (self-selection / self-organisation / choice from a speciCed listing)

Main learning outcomes and themes

Short-answer text

Integration of SSC assessments with the remainder of the curriculum

Percentage of the overall assessment that is dedicated to SSC

Please, indicate the URL if you have an open access webpage that describes your SSC programme

Have there been any significant changes over the last 5 years? If yes, what?

### Career exploration

Is exploring career options one of the objectives of your SSC programme? If yes, do you think it is working?

Do you think your SSC programme influences future career choice?

SSCs are considered to be an opportunity to students to explore potential careers. Do you think your programme effectively does this? Please justify your answer.

Do you think your programme could be more effectively designed to offer students better opportunities for career exploration? Please justify your answer.

Do you think your programme could be more effectively designed to offer students better opportunities for career exploration? Please justify your answer.

### SSC network

Do you feel isolated as SSC lead?

Do you think an SSC network would be useful? Please justify your answer.

Thank you very much for your contribution
