# Supplementary material for: Influence of Student Selected Components on Medical Career Choice
Source: MedEdPublish (2016). 2017 Oct 26;6:191. [Version 1] doi: 10.15694/mep.2017.000191 (PMC10885311; doi:10.15694/mep.2017.000191)
Supplement: Peer review history

# Reviewer report: Reviewer response for version 1

**DOI:** 10.21956/mep.19378.r28392

**Published:** 2017-11-25

**Authors:** Barbara Jennings

**Affiliations:** 1 Norwich Medical School

This review has been migrated. The reviewer awarded 4 stars out of 5 Like the other reviewers, I would also recommend this paper to the faculty teams responsible for delivering SSC programmes at medical schools. I was particularly interested to hear the reflections of recent medical graduates about whether these curricular opportunities had a role in shaping their career choices.One of the challenges, for SSC programme leads, is that this part of the curriculum has objectives beyond career exploration, including in depth study of a topic of choice, or opportunity to undertake research. Interestingly, an SSC attachment with a clinical or laboratory research team can have a strong influence on career paths because it can result in decisions (both positive & negative) about intercalation, and in the UK it may influence application for academic-FY2 places.The shape and scope of SSC programmes may also reflect the medical course design; for example, we describe the longitudinal student-selected-studies part of our programme at Norwich Medical School (NMS) in a case study in this journal https://www.mededpublish.org/manuscripts/372 (n.b. possible COI, I was a co-author for this paper). Its design is complementary to a systems-based modular course but may not fit with a traditional degree. It would be interesting to ask SSC leads about this. The authors carefully discuss a number of limitations for their study, but don’t elaborate on the reason behind the gaps in data in the supplemental table in the appendix. Also, the approach used to systematically identify the required website material and medical school leads is not described in the methods section. I was sorry not to see a full summary of the NMS programme, and if the authors are continuing with this work I can put them in touch with the responsible faculty members.

Reviewer Expertise:

NA

No decision status is available

# Reviewer report: Reviewer response for version 1

**DOI:** 10.21956/mep.19378.r28391

**Published:** 2017-11-07

**Authors:** Susmita Reddy Karri

**Affiliations:** 1 Topiwala national medical college and BYL nair charitable hospital, mumbai. india

This review has been migrated. The reviewer awarded 4 stars out of 5 I like the way in which the views of FY2 doctors' and the medical schools' views are out forth and it is interesting to note their difference in opinion.My only point of concern are the figures which show different number of total students. I presume it is because some of the questions were not answered by the students. Would like to have more clarity in this regard.

Reviewer Expertise:

NA

No decision status is available

# Reviewer report: Reviewer response for version 1

**DOI:** 10.21956/mep.19378.r28390

**Published:** 2017-10-28

**Authors:** Megan Anakin

**Affiliations:** 1 University of Otago

This review has been migrated. The reviewer awarded 4 stars out of 5 I recommend that this article be read and discussed with colleagues. Even though this article is focused on the UK context of student selective components, the topic of preparing finishing students and junior healthcare professionals to make career choices is relevant to educators in the health professions globally. The important limitations to this study were well described by the authors.I have a few suggestions about the presentation of the results to make this article easier to read:• In the second paragraph that reports “The students’ perspective: FY2 doctor’s questionnaire”, a table showing frequencies and percentages may present the overall results more clearly to a reader than descriptions. • Figures 1 to 4 are presented very briefly in one paragraph and the reader is left to make sense of each figure. It would be helpful to direct the attention of the reader to the one or two key findings in presented in each Figure It would also be helpful to state and explain a little more about the sample illustrated in each Figure. For example, in Figure 1, n = 71 and in Figure 2, n = 30. If Figures 1 and 2 are supposed to account for all participants, I am left wondering what responses did the 2 missing participants give or were they excluded for some reason? In Figure 3, n = 65 and in Figure 4, n = 59. Again, I’m wondering why responses from all 103 participants were not represented? • By addressing the suggestion above, it will help to eliminate one source of confusion for me involving the discrepancy between the statements “75% mentioned that they would have liked to have further opportunities” and “Their suggestions are summarised in Figure 4” where only n = 59 or 57% of participants' suggestions are represented.

Reviewer Expertise:

NA

No decision status is available

# Reviewer report: Reviewer response for version 1

**DOI:** 10.21956/mep.19378.r28389

**Published:** 2017-10-27

**Authors:** Trevor Gibbs

**Affiliations:** 1 AMEE

This review has been migrated. The reviewer awarded 5 stars out of 5 Although the original plan for SSCs ( or SSMs as they were originally described) was to support a core plus options change to the previous styles of curricula structure, the concept soon changed with the second version of Tomorrow's Doctors and included a requirement for schools to "think" about future medical careers. It would appear from observation that this element has become quite important now; hence the importance of this paper. Despite the low numbers of examples drawn from the UK schools (for a reason which itself needs investigation), this well-written paper promotes a strong point for re-alignment of SSCs, and several very useful ideas to consider of how SSCs now fit in with curricula and career needs. I would strongly recommend this paper for all those faculty involved in SSCs and particularly draw their attention to the implied need of re-visiting the learning outcomes of their SSCs.

Reviewer Expertise:

NA

No decision status is available
